# *N*-Benzyl­idenenordehydro­abietylamine

**DOI:** 10.1107/S1600536809022909

**Published:** 2009-06-20

**Authors:** Xiao-Ping Rao, Yong Wu, Zhan-Qian Song, Shi-Bin Shang

**Affiliations:** a Institute of Chemical Industry of Forest Products, Chinese Academy of Forestry, Nanjing 210042, People’s Republic of China

## Abstract

The title compound [systematic name: (1*R*,4a*S*,10a*R*,*E*)-*N*-benzyl­idene-7-isopropyl-1,4a-dimethyl-1,2,3,4,4a,9,10,10a-octa­hydro­phenanthren-1-amine], C_26_H_33_N, has been synthesized from nor-dehydro­abietylamine and benzaldehyde. The two cyclo­hexane rings form a *trans* ring junction with classic chair and half-chair conformations, respectively, the two methyl groups are on the same side of tricyclic hydro­phenanthrene structure. The dihedral angle between two benzene rings is 44.2 (4)°. The C=N bond is in an *E* configuration.

## Related literature

For the biological activity of dehydro­abietiylamine derivatives, see: Rao *et al.* (2006 ▶); Rao, Song & He (2008 ▶); Rao, Song, He & Jia (2008 ▶); Wilkerson *et al.* (1993 ▶).
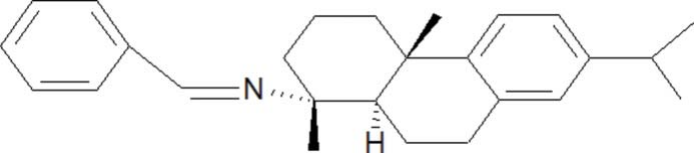


## Experimental

### 

#### Crystal data

C_26_H_33_N
*M* *_r_* = 359.53
Monoclinic, 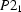

*a* = 12.285 (3) Å
*b* = 5.8940 (12) Å
*c* = 14.994 (3) Å
β = 95.90 (3)°
*V* = 1079.9 (4) Å^3^
*Z* = 2
Mo *K*α radiation
μ = 0.06 mm^−1^
*T* = 293 K
0.40 × 0.30 × 0.30 mm

#### Data collection

Enraf–Nonius CAD-4 diffractometer
Absorption correction: multi-scan (*SADABS*; Sheldrick, 1996 ▶) *T*_min_ = 0.975, *T*_max_ = 0.981
2435 measured reflections
2324 independent reflections
1761 reflections with *I* > 2σ(*I*)
*R*_int_ = 0.044
3 standard reflections every 200 reflections intensity decay: none

#### Refinement

*R*[*F* ^2^ > 2σ(*F* ^2^)] = 0.074
*wR*(*F* ^2^) = 0.199
*S* = 1.04
2324 reflections
244 parameters
2 restraints
H-atom parameters constrained
Δρ_max_ = 0.30 e Å^−3^
Δρ_min_ = −0.36 e Å^−3^

### 

Data collection: *CAD-4 Software* (Enraf–Nonius, 1989 ▶); cell refinement: *CAD-4 Software*; data reduction: *XCAD4* (Harms & Wocadlo, 1995 ▶); program(s) used to solve structure: *SHELXS97* (Sheldrick, 2008 ▶); program(s) used to refine structure: *SHELXL97* (Sheldrick, 2008 ▶); molecular graphics: *SHELXTL* (Sheldrick, 2008 ▶); software used to prepare material for publication: *SHELXTL*.

## Supplementary Material

Crystal structure: contains datablocks I, global. DOI: 10.1107/S1600536809022909/at2811sup1.cif

Structure factors: contains datablocks I. DOI: 10.1107/S1600536809022909/at2811Isup2.hkl

Additional supplementary materials:  crystallographic information; 3D view; checkCIF report

## Figures and Tables

**Fig. 1.**
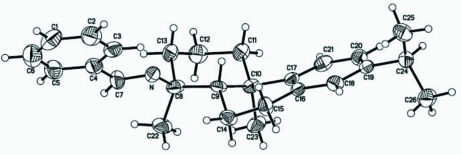
The molecular structure of the title compound, with H atoms represented by small spheres of arbitrary radius and displacement ellipsoids at the 30% probability level.

## supplementary crystallographic information

### Comment

Dehydroabietiylamine derivatives exhibit wide range of biological activities, such as antifungal and antitumor activity (Wilkerson *et al.*, 1993 and Rao *et al.*, 2006). Nor-dehydroabietylamine is a new derivative of dehydoabietylamine, which the amine group directly attached to the tricyclic hydrophenthranene structure (Rao *et al.*, 2006). Although much attention has been paid to the bioactivity of dehydroabietylamine derivatives, the crystal structure of the title compound has not yet been reported. In this work, we describe the crystal structure of the title compound.

As shown in Fig. 1, the title compound contains four rings, the two cyclohexane rings form a *trans* ring junction with classic chair and half-chair conformation, respectively, the two methyl groups are in the axis position of the cyclohexane ring. The two benzene rings are almost planar, the dihedral angle between them is 44.2 °. The bond lengths and bond angles in the molecule are in normal ranges. The title structure is compared with previously found structure 4-chloro-2-((*E*)-(((1*R*,4aS,10aR)-7-isopropyl-1,4a-dimethyl- 1,2,3,4,4a,9,10,10*a*-octahydrophenanthren-1-yl) methylimino)methyl)phenol (Rao *et al.*,2006). They exhibited almost the same configurations except that the imine group directly attached to the hydrophenanthrene structure of the title structure.

### Experimental

A mixture of nor-dehydroabietylamine (1 mmol), benzaldehyde (1 mmol) and ethanol (20 ml) was stirred at 353 K for 4 h, then the solvent was distilled off. Upon recrystallization from acetone, white crystals of the title compound were obtained. Single crystals were grown from acetone.

### Refinement

H atoms were positioned geometrically and refined as riding atoms, with C—H = 0.96Å and *U*_iso_(H) = 1.5*U*_eq_(C) for methyl H atoms, and C—H = 0.937–0.98Å and *U*_iso_(H) = 1.2*U*_eq_(C) for all other H atoms. In the absence of significant anomalous scattering effects, Friedel pairs were merged.

### Crystal data

**Table:**

C_26_H_33_N	*F*(000) = 392
*M**_r_* = 359.53	*D*_x_ = 1.106 Mg m^−^^3^
Monoclinic, *P*2_1_	Mo *K*α radiation, λ = 0.71073 Å
Hall symbol: P 2yb	Cell parameters from 25 reflections
*a* = 12.285 (3) Å	θ = 10–13°
*b* = 5.8940 (12) Å	µ = 0.06 mm^−^^1^
*c* = 14.994 (3) Å	*T* = 293 K
β = 95.90 (3)°	Block, white
*V* = 1079.9 (4) Å^3^	0.40 × 0.30 × 0.30 mm
*Z* = 2	

### Data collection

**Table:**

Enraf–Nonius CAD-4 diffractometer	1761 reflections with *I* > 2σ(*I*)
Radiation source: fine-focus sealed tube	*R*_int_ = 0.044
graphite	θ_max_ = 26.0°, θ_min_ = 1.4°
ω/2θ scans	*h* = −15→15
Absorption correction: multi-scan (*SADABS*; Sheldrick, 1996)	*k* = 0→7
*T*_min_ = 0.975, *T*_max_ = 0.981	*l* = 0→18
2435 measured reflections	3 standard reflections every 200 reflections
2324 independent reflections	intensity decay: none

### Refinement

**Table:**

Refinement on *F*^2^	Primary atom site location: structure-invariant direct methods
Least-squares matrix: full	Secondary atom site location: difference Fourier map
*R*[*F*^2^ > 2σ(*F*^2^)] = 0.074	Hydrogen site location: inferred from neighbouring sites
*wR*(*F*^2^) = 0.199	H-atom parameters constrained
*S* = 1.04	*w* = 1/[σ^2^(*F*_o_^2^) + (0.03*P*)^2^ + 2.5*P*] where *P* = (*F*_o_^2^ + 2*F*_c_^2^)/3
2324 reflections	(Δ/σ)_max_ < 0.001
244 parameters	Δρ_max_ = 0.30 e Å^−^^3^
2 restraints	Δρ_min_ = −0.36 e Å^−^^3^

### Special details

**Table:**

Experimental. Although the absolute configuration could not be determined in this case, it has been determined in our previous article which indicated the chiral centers exhibited R,S and R configurations, respectively.
Geometry. All e.s.d.'s (except the e.s.d. in the dihedral angle between two l.s. planes) are estimated using the full covariance matrix. The cell e.s.d.'s are taken into account individually in the estimation of e.s.d.'s in distances, angles and torsion angles; correlations between e.s.d.'s in cell parameters are only used when they are defined by crystal symmetry. An approximate (isotropic) treatment of cell e.s.d.'s is used for estimating e.s.d.'s involving l.s. planes.
Refinement. Refinement of *F*^2^ against ALL reflections. The weighted *R*-factor *wR* and goodness of fit *S* are based on *F*^2^, conventional *R*-factors *R* are based on *F*, with *F* set to zero for negative *F*^2^. The threshold expression of *F*^2^ > σ(*F*^2^) is used only for calculating *R*-factors(gt) *etc*. and is not relevant to the choice of reflections for refinement. *R*-factors based on *F*^2^ are statistically about twice as large as those based on *F*, and *R*- factors based on ALL data will be even larger.

### Fractional atomic coordinates and isotropic or equivalent isotropic displacement parameters (Å^2^)

**Table:**

	*x*	*y*	*z*	*U*_iso_*/*U*_eq_	
N	0.5943 (4)	0.1064 (10)	0.6722 (3)	0.0555 (15)	
C1	0.9610 (7)	−0.212 (2)	0.6297 (5)	0.083 (3)	
H1A	1.0249	−0.2959	0.6287	0.100*	
C2	0.8832 (7)	−0.2846 (18)	0.6812 (5)	0.080 (2)	
H2A	0.8933	−0.4180	0.7142	0.096*	
C3	0.7893 (7)	−0.1591 (15)	0.6841 (4)	0.067 (2)	
H3A	0.7358	−0.2059	0.7197	0.080*	
C4	0.7749 (5)	0.0366 (14)	0.6340 (4)	0.061 (2)	
C5	0.8544 (7)	0.1063 (16)	0.5820 (5)	0.072 (2)	
H5A	0.8454	0.2385	0.5482	0.087*	
C6	0.9483 (7)	−0.024 (2)	0.5807 (6)	0.091 (3)	
H6A	1.0027	0.0210	0.5455	0.109*	
C7	0.6712 (5)	0.1704 (15)	0.6342 (4)	0.065 (2)	
H7A	0.6656	0.3083	0.6040	0.078*	
C8	0.4875 (7)	0.2395 (12)	0.6683 (4)	0.0573 (19)	
C9	0.4313 (5)	0.1432 (11)	0.7462 (3)	0.0460 (15)	
H9A	0.4370	−0.0218	0.7401	0.055*	
C10	0.3056 (5)	0.1900 (11)	0.7458 (4)	0.0436 (14)	
C11	0.2496 (5)	0.1195 (14)	0.6530 (4)	0.0552 (17)	
H11A	0.1732	0.1639	0.6486	0.066*	
H11B	0.2524	−0.0443	0.6476	0.066*	
C12	0.3039 (6)	0.2281 (15)	0.5748 (4)	0.068 (2)	
H12A	0.2670	0.1766	0.5181	0.082*	
H12B	0.2970	0.3919	0.5774	0.082*	
C13	0.4198 (5)	0.1662 (15)	0.5803 (4)	0.0613 (19)	
H13A	0.4517	0.2347	0.5302	0.074*	
H13B	0.4254	0.0029	0.5743	0.074*	
C14	0.4894 (5)	0.1957 (14)	0.8384 (4)	0.0567 (17)	
H14A	0.5680	0.1840	0.8368	0.068*	
H14B	0.4726	0.3494	0.8556	0.068*	
C15	0.4526 (5)	0.0303 (16)	0.9058 (4)	0.061 (2)	
H15A	0.4719	0.0913	0.9654	0.073*	
H15B	0.4928	−0.1102	0.9015	0.073*	
C16	0.3317 (4)	−0.0234 (11)	0.8955 (3)	0.0389 (13)	
C17	0.2618 (5)	0.0468 (10)	0.8206 (3)	0.0412 (14)	
C18	0.2886 (5)	−0.1458 (12)	0.9632 (4)	0.0501 (16)	
H18A	0.3349	−0.1858	1.0137	0.060*	
C19	0.1794 (5)	−0.2108 (11)	0.9587 (4)	0.0452 (14)	
C20	0.1139 (5)	−0.1380 (13)	0.8849 (4)	0.0527 (17)	
H20A	0.0400	−0.1755	0.8800	0.063*	
C21	0.1526 (5)	−0.0118 (13)	0.8179 (4)	0.0544 (17)	
H21A	0.1043	0.0352	0.7697	0.065*	
C22	0.5043 (8)	0.4935 (13)	0.6724 (5)	0.079 (3)	
H22A	0.4345	0.5680	0.6689	0.118*	
H22B	0.5428	0.5409	0.6231	0.118*	
H22C	0.5462	0.5328	0.7279	0.118*	
C23	0.2709 (7)	0.4386 (12)	0.7645 (5)	0.068 (2)	
H23A	0.1926	0.4468	0.7627	0.103*	
H23B	0.2952	0.5373	0.7196	0.103*	
H23C	0.3034	0.4848	0.8226	0.103*	
C24	0.1371 (6)	−0.3531 (14)	1.0319 (4)	0.0597 (19)	
H24A	0.0603	−0.3865	1.0122	0.072*	
C25	0.1949 (7)	−0.5788 (13)	1.0437 (6)	0.080 (3)	
H25A	0.1657	−0.6626	1.0908	0.120*	
H25B	0.2717	−0.5541	1.0592	0.120*	
H25C	0.1839	−0.6633	0.9888	0.120*	
C26	0.1383 (7)	−0.2230 (16)	1.1198 (4)	0.082 (3)	
H26A	0.1122	−0.3194	1.1646	0.123*	
H26B	0.0919	−0.0922	1.1111	0.123*	
H26C	0.2117	−0.1755	1.1392	0.123*	

### Atomic displacement parameters (Å^2^)

**Table:**

	*U*^11^	*U*^22^	*U*^33^	*U*^12^	*U*^13^	*U*^23^
N	0.066 (3)	0.048 (3)	0.056 (3)	−0.002 (3)	0.022 (3)	−0.004 (3)
C1	0.070 (5)	0.115 (9)	0.063 (5)	0.016 (6)	0.002 (4)	−0.023 (6)
C2	0.100 (6)	0.076 (6)	0.063 (4)	−0.008 (6)	0.004 (4)	0.004 (5)
C3	0.087 (5)	0.065 (5)	0.048 (4)	−0.012 (5)	0.006 (4)	−0.012 (4)
C4	0.067 (4)	0.070 (5)	0.044 (3)	−0.018 (4)	−0.001 (3)	−0.007 (4)
C5	0.088 (5)	0.068 (6)	0.062 (4)	−0.010 (5)	0.011 (4)	0.007 (4)
C6	0.075 (6)	0.127 (10)	0.069 (5)	−0.022 (7)	0.003 (4)	0.000 (7)
C7	0.070 (4)	0.073 (5)	0.055 (4)	−0.017 (4)	0.020 (3)	0.004 (4)
C8	0.095 (5)	0.036 (4)	0.041 (3)	−0.001 (4)	0.008 (3)	−0.003 (3)
C9	0.076 (4)	0.030 (3)	0.031 (3)	−0.013 (3)	0.000 (3)	−0.001 (3)
C10	0.061 (4)	0.033 (3)	0.036 (3)	0.011 (3)	0.002 (2)	0.004 (3)
C11	0.062 (4)	0.063 (5)	0.040 (3)	0.005 (4)	0.002 (3)	0.006 (3)
C12	0.096 (5)	0.066 (5)	0.040 (3)	−0.002 (5)	0.000 (3)	0.013 (4)
C13	0.078 (4)	0.073 (5)	0.034 (3)	−0.012 (5)	0.012 (3)	0.004 (4)
C14	0.067 (4)	0.056 (4)	0.044 (3)	−0.014 (4)	−0.005 (3)	−0.001 (3)
C15	0.054 (4)	0.091 (6)	0.038 (3)	−0.022 (4)	0.000 (3)	0.005 (4)
C16	0.044 (3)	0.038 (3)	0.035 (3)	0.000 (3)	0.005 (2)	−0.003 (3)
C17	0.059 (4)	0.035 (3)	0.031 (3)	0.001 (3)	0.008 (2)	−0.002 (3)
C18	0.060 (4)	0.051 (4)	0.037 (3)	−0.003 (3)	−0.006 (3)	0.005 (3)
C19	0.057 (3)	0.040 (4)	0.041 (3)	−0.002 (3)	0.012 (3)	−0.007 (3)
C20	0.046 (3)	0.063 (5)	0.048 (3)	−0.004 (4)	0.003 (3)	−0.008 (4)
C21	0.063 (4)	0.058 (5)	0.041 (3)	0.013 (4)	−0.004 (3)	−0.004 (3)
C22	0.134 (8)	0.036 (4)	0.069 (5)	−0.022 (5)	0.022 (5)	0.004 (4)
C23	0.105 (6)	0.037 (4)	0.068 (4)	0.019 (4)	0.030 (4)	0.001 (4)
C24	0.062 (4)	0.067 (5)	0.052 (3)	−0.015 (4)	0.015 (3)	−0.006 (4)
C25	0.109 (6)	0.038 (4)	0.099 (6)	−0.006 (5)	0.031 (5)	0.010 (4)
C26	0.135 (7)	0.069 (6)	0.045 (4)	−0.015 (6)	0.024 (4)	0.005 (4)

### Geometric parameters (Å, °)

**Table:**

N—C7	1.212 (7)	C14—C15	1.508 (9)
N—C8	1.524 (9)	C14—H14A	0.9700
C1—C6	1.331 (14)	C14—H14B	0.9700
C1—C2	1.359 (11)	C15—C16	1.510 (8)
C1—H1A	0.9300	C15—H15A	0.9700
C2—C3	1.375 (11)	C15—H15B	0.9700
C2—H2A	0.9300	C16—C18	1.393 (8)
C3—C4	1.378 (11)	C16—C17	1.405 (7)
C3—H3A	0.9300	C17—C21	1.381 (8)
C4—C5	1.374 (9)	C18—C19	1.389 (8)
C4—C7	1.499 (7)	C18—H18A	0.9300
C5—C6	1.387 (12)	C19—C20	1.368 (8)
C5—H5A	0.9300	C19—C24	1.515 (9)
C6—H6A	0.9300	C20—C21	1.374 (9)
C7—H7A	0.9300	C20—H20A	0.9300
C8—C22	1.512 (10)	C21—H21A	0.9300
C8—C9	1.525 (9)	C22—H22A	0.9600
C8—C13	1.547 (9)	C22—H22B	0.9600
C9—C14	1.522 (7)	C22—H22C	0.9600
C9—C10	1.568 (8)	C23—H23A	0.9600
C9—H9A	0.9800	C23—H23B	0.9600
C10—C17	1.544 (8)	C23—H23C	0.9600
C10—C11	1.545 (8)	C24—C25	1.509 (11)
C10—C23	1.559 (9)	C24—C26	1.524 (10)
C11—C12	1.546 (9)	C24—H24A	0.9800
C11—H11A	0.9700	C25—H25A	0.9600
C11—H11B	0.9700	C25—H25B	0.9600
C12—C13	1.464 (9)	C25—H25C	0.9600
C12—H12A	0.9700	C26—H26A	0.9600
C12—H12B	0.9700	C26—H26B	0.9600
C13—H13A	0.9700	C26—H26C	0.9600
C13—H13B	0.9700		
			
C7—N—C8	122.1 (7)	C15—C14—H14A	109.8
C6—C1—C2	121.9 (10)	C9—C14—H14A	109.8
C6—C1—H1A	119.1	C15—C14—H14B	109.8
C2—C1—H1A	119.1	C9—C14—H14B	109.8
C1—C2—C3	119.3 (10)	H14A—C14—H14B	108.2
C1—C2—H2A	120.4	C14—C15—C16	115.3 (5)
C3—C2—H2A	120.4	C14—C15—H15A	108.5
C2—C3—C4	119.7 (8)	C16—C15—H15A	108.5
C2—C3—H3A	120.2	C14—C15—H15B	108.5
C4—C3—H3A	120.2	C16—C15—H15B	108.5
C5—C4—C3	120.0 (7)	H15A—C15—H15B	107.5
C5—C4—C7	119.9 (7)	C18—C16—C17	119.2 (5)
C3—C4—C7	120.1 (7)	C18—C16—C15	118.5 (5)
C4—C5—C6	119.0 (8)	C17—C16—C15	122.3 (5)
C4—C5—H5A	120.5	C21—C17—C16	117.5 (5)
C6—C5—H5A	120.5	C21—C17—C10	121.7 (5)
C1—C6—C5	120.2 (9)	C16—C17—C10	120.8 (5)
C1—C6—H6A	119.9	C19—C18—C16	123.0 (5)
C5—C6—H6A	119.9	C19—C18—H18A	118.5
N—C7—C4	122.8 (8)	C16—C18—H18A	118.5
N—C7—H7A	118.6	C20—C19—C18	115.9 (6)
C4—C7—H7A	118.6	C20—C19—C24	122.8 (6)
C22—C8—N	113.3 (7)	C18—C19—C24	121.3 (6)
C22—C8—C9	114.1 (6)	C19—C20—C21	122.8 (6)
N—C8—C9	103.6 (5)	C19—C20—H20A	118.6
C22—C8—C13	111.7 (7)	C21—C20—H20A	118.6
N—C8—C13	105.9 (5)	C20—C21—C17	121.5 (6)
C9—C8—C13	107.6 (6)	C20—C21—H21A	119.3
C14—C9—C8	114.3 (5)	C17—C21—H21A	119.3
C14—C9—C10	109.7 (5)	C8—C22—H22A	109.5
C8—C9—C10	117.1 (5)	C8—C22—H22B	109.5
C14—C9—H9A	104.8	H22A—C22—H22B	109.5
C8—C9—H9A	104.8	C8—C22—H22C	109.5
C10—C9—H9A	104.8	H22A—C22—H22C	109.5
C17—C10—C11	110.6 (5)	H22B—C22—H22C	109.5
C17—C10—C23	105.2 (5)	C10—C23—H23A	109.5
C11—C10—C23	108.1 (6)	C10—C23—H23B	109.5
C17—C10—C9	108.5 (5)	H23A—C23—H23B	109.5
C11—C10—C9	107.6 (5)	C10—C23—H23C	109.5
C23—C10—C9	116.9 (6)	H23A—C23—H23C	109.5
C10—C11—C12	112.6 (6)	H23B—C23—H23C	109.5
C10—C11—H11A	109.1	C25—C24—C19	112.4 (6)
C12—C11—H11A	109.1	C25—C24—C26	112.2 (7)
C10—C11—H11B	109.1	C19—C24—C26	112.0 (6)
C12—C11—H11B	109.1	C25—C24—H24A	106.6
H11A—C11—H11B	107.8	C19—C24—H24A	106.6
C13—C12—C11	110.3 (6)	C26—C24—H24A	106.6
C13—C12—H12A	109.6	C24—C25—H25A	109.5
C11—C12—H12A	109.6	C24—C25—H25B	109.5
C13—C12—H12B	109.6	H25A—C25—H25B	109.5
C11—C12—H12B	109.6	C24—C25—H25C	109.5
H12A—C12—H12B	108.1	H25A—C25—H25C	109.5
C12—C13—C8	114.4 (6)	H25B—C25—H25C	109.5
C12—C13—H13A	108.7	C24—C26—H26A	109.5
C8—C13—H13A	108.7	C24—C26—H26B	109.5
C12—C13—H13B	108.7	H26A—C26—H26B	109.5
C8—C13—H13B	108.7	C24—C26—H26C	109.5
H13A—C13—H13B	107.6	H26A—C26—H26C	109.5
C15—C14—C9	109.4 (5)	H26B—C26—H26C	109.5
			
C6—C1—C2—C3	−1.0 (13)	C22—C8—C13—C12	−71.4 (9)
C1—C2—C3—C4	0.9 (12)	N—C8—C13—C12	164.8 (7)
C2—C3—C4—C5	−0.5 (10)	C9—C8—C13—C12	54.6 (9)
C2—C3—C4—C7	177.9 (7)	C8—C9—C14—C15	−160.1 (6)
C3—C4—C5—C6	0.3 (11)	C10—C9—C14—C15	66.1 (7)
C7—C4—C5—C6	−178.2 (7)	C9—C14—C15—C16	−41.1 (8)
C2—C1—C6—C5	0.8 (14)	C14—C15—C16—C18	−170.0 (6)
C4—C5—C6—C1	−0.4 (13)	C14—C15—C16—C17	9.6 (9)
C8—N—C7—C4	−177.2 (6)	C18—C16—C17—C21	−0.4 (9)
C5—C4—C7—N	173.2 (7)	C15—C16—C17—C21	179.9 (6)
C3—C4—C7—N	−5.3 (11)	C18—C16—C17—C10	177.8 (6)
C7—N—C8—C22	−38.6 (9)	C15—C16—C17—C10	−1.9 (9)
C7—N—C8—C9	−162.7 (6)	C11—C10—C17—C21	−39.0 (8)
C7—N—C8—C13	84.1 (8)	C23—C10—C17—C21	77.4 (8)
C22—C8—C9—C14	−56.7 (9)	C9—C10—C17—C21	−156.8 (6)
N—C8—C9—C14	66.8 (7)	C11—C10—C17—C16	142.8 (6)
C13—C8—C9—C14	178.7 (6)	C23—C10—C17—C16	−100.8 (7)
C22—C8—C9—C10	73.6 (9)	C9—C10—C17—C16	25.0 (7)
N—C8—C9—C10	−162.9 (5)	C17—C16—C18—C19	2.7 (10)
C13—C8—C9—C10	−51.0 (7)	C15—C16—C18—C19	−177.6 (7)
C14—C9—C10—C17	−56.7 (7)	C16—C18—C19—C20	−3.0 (10)
C8—C9—C10—C17	170.8 (5)	C16—C18—C19—C24	177.1 (7)
C14—C9—C10—C11	−176.4 (6)	C18—C19—C20—C21	1.2 (10)
C8—C9—C10—C11	51.2 (7)	C24—C19—C20—C21	−179.0 (7)
C14—C9—C10—C23	61.9 (7)	C19—C20—C21—C17	1.0 (11)
C8—C9—C10—C23	−70.5 (7)	C16—C17—C21—C20	−1.3 (10)
C17—C10—C11—C12	−170.3 (6)	C10—C17—C21—C20	−179.5 (6)
C23—C10—C11—C12	75.1 (7)	C20—C19—C24—C25	120.6 (7)
C9—C10—C11—C12	−51.9 (7)	C18—C19—C24—C25	−59.6 (9)
C10—C11—C12—C13	58.1 (9)	C20—C19—C24—C26	−112.0 (8)
C11—C12—C13—C8	−59.1 (9)	C18—C19—C24—C26	67.9 (8)
